# Ferroptosis and its emerging role in esophageal cancer

**DOI:** 10.3389/fmolb.2022.1027912

**Published:** 2022-09-27

**Authors:** Rezeye Maimaitizunong, Kai Wang, Hui Li

**Affiliations:** ^1^ Department of Biochemistry and Molecular Biology, Basic Medicine School, Xinjiang Medical University, Urumqi, China; ^2^ Department of Medical Engineering and Technology, Xinjiang Medical University, Urumqi, China; ^3^ Central Laboratory of Xinjiang Medical University, Urumqi, China

**Keywords:** ferroptosis, lipid peroxidation, antioxidant system, esophageal cancer, non-coding RNA

## Abstract

The occurrence and development of tumors involve a series of life activities of cells, among which cell death has always been a crucial part in the research of tumor mechanisms and treatment methods. Ferroptosis is a non-apoptotic form of cell death, which is characterized by lipid peroxidation accumulation and further cell membrane rupture caused by excessive production of intracellular oxygen free radicals dependent on iron ions. Esophageal cancer is one of the common digestive tract tumors. Patients in the early stage are mainly treated with surgery, and the curative effect is awe-inspiring. However, surgery is far from enough for terminal patients, and it is the best choice to combine radiotherapy and chemotherapy before the operation or during the perioperative period. Although the treatment plan for patients with advanced esophageal cancer is constantly being optimized, we are disappointed at the still meager 5-year survival rate of patients and the poor quality of life. A series of complex problems, such as increased chemotherapy drug resistance and decreased radiotherapy sensitivity of esophageal cancer cells, are waiting for us to tackle. Perhaps ferroptosis can provide practical and feasible solutions and bring new hope to patients with advanced esophageal cancer. The occurrence of ferroptosis is related to the dysregulation of iron metabolism, lipid metabolism, and glutamate metabolism. Therefore, these dysregulated metabolic participant proteins and signaling pathways are essential entry points for using cellular ferroptosis to resist the occurrence and development of cancer cells. This review first introduced the main regulatory mechanisms of ferroptosis. It then summarized the current research status of ferroptosis in esophageal cancer, expecting to provide ideas for the research related to ferroptosis in esophageal cancer.

## Introduction

**FIGURE 1:**
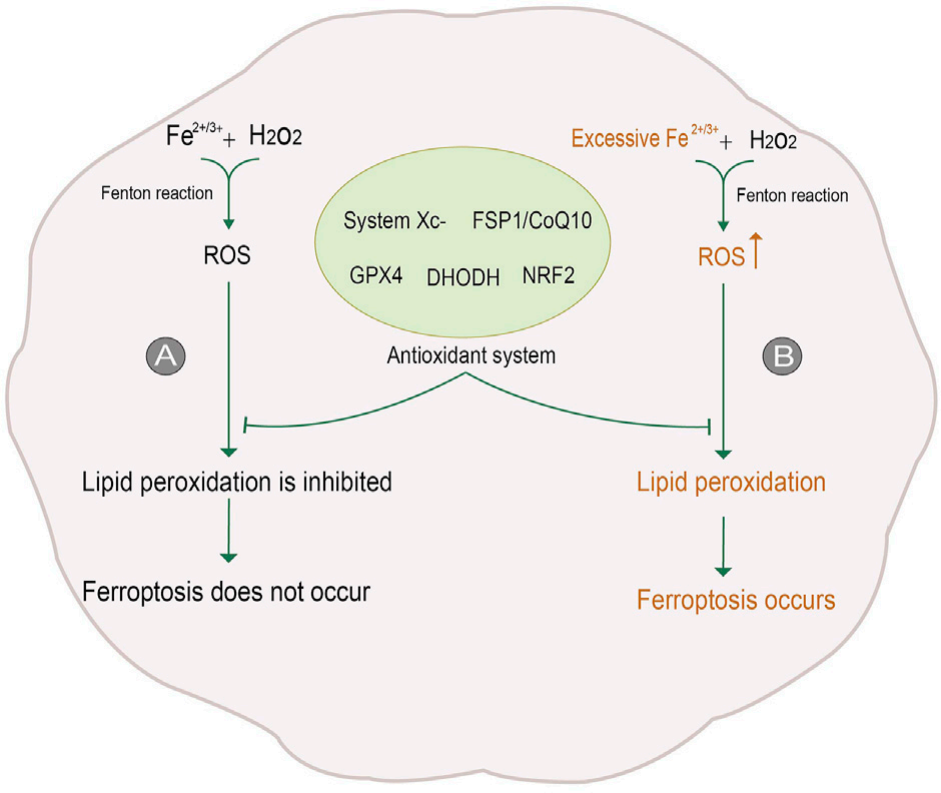
Process of ferroptosis. **(A)** In general, Reactive Oxygen Species (ROS) is generated by iron ions and hydrogen peroxide in cells through the Fenton reaction. ROS is removed under the action of the antioxidant system, inhibiting lipid peroxidation and thus avoiding ferroptosis. **(B)** When intracellular iron levels rise, excessive ROS will be produced. The antioxidant system is not enough to counteract the excessive ROS, so lipid peroxidation is not inhibited, and ferroptosis occurs. The figure modified from (Yan et al., 2021).

Cell death is a significant step in organism growth and development. Moreover, different forms of cell death are regulated differently; imbalanced regulation could be the first step in the occurrence and development of diseases. Traditionally, cell death can be classified into apoptosis, necrosis, and autophagy according to the change in mechanism and cell morphology (Kroemer et al., 2009). With the in-depth study of cell death, a non-apoptotic form of cell death, namely ferroptosis, was first proposed in 2012. Ferroptosis is a process of oxidative damage after the accumulation of oxygen free radicals dependent on iron ions. Excessive iron ions in cells react with hydrogen peroxide to produce reactive Oxygen Species (ROS). Excessive ROS will attack polyunsaturated fatty acid (PUFA) in biomembrane to deprive hydrogen ions, making it into a peroxide state. Eventually, the lipids in cell membranes and intracellular organelles become lipid peroxide and rupture due to massive peroxidation, resulting in cell death (Dixon et al., 2012). In general, there are many sources of oxygen free radicals in cells, to eliminate these oxygen radicals, cells contain potent antioxidant mechanisms. Despite the protective mechanism, when too many oxygen radicals are produced, the balance is disrupted, and oxidative damage can occur, leading to further cell death (Jamar et al., 2017) (Figure 1).

The ferroptosis process is a complex and sophisticated life phenomenon that occurs under the joint control of multiple systems, multiple metabolism, and multiple genes. According to the data provided by the FerrDb database, 259 genes are involved in ferroptosis. Based on their roles in ferroptosis, they can be divided into ferroptosis driver genes, ferroptosis suppressor genes, and ferroptosis marker genes. Among them were 108 driver genes, 69 repressor genes and 111 marker genes. The total number of the three genes was significantly higher than 259. The reason was that 28 genes had multiple identities in the ferroptosis. These genes have become the focus of research on disease mechanisms. In malignant tumors, ferroptosis can be triggered to eliminate tumor cells, while inhibiting this process can prevent acute damage to normal cells and neurodegenerative diseases. For example, Erastin (ferroptosis inducer) was used to activate the ferroptosis pathway in acute myelogenous leukemia cells, which was found to be more sensitive to chemotherapy agents (Yu et al., 2015); Treatment of breast cancer cells with Siramesine and lapatinib enhanced the occurrence of ferroptosis (Ma et al., 2016); Sorafenib (an oncogenic kinase inhibitor) can effectively induce ferroptosis in different solid tumor cell lines (Lachaier et al., 2014). Among non-tumor diseases: the combined treatment of iron inhibin -1 and iron chelating agent (both ferroptosis inhibitors) significantly improved the heart failure of mice (Fang et al., 2019); After the mice with intracerebral hemorrhage were treated with iron inhibin -1, the neurological deficit, memory impairment and brain atrophy caused by intracerebral bleeding were significantly reduced (Chen et al., 2019). After treatment with liproxstatin-1 (ferroptosis inhibitor), liver lipid peroxidation and related cell death were obviously inhibited, thus reducing the severity of nonalcoholic steatohepatitis (Qi et al., 2020). In addition, more research results show that ferroptosis is an essential participant in disease occurrence and development, as well as malignant tumor deterioration and metastasis.

Since infinite proliferation and malignant metastasis are two characteristics of malignant tumors and two major problems in clinical treatment, inducing the death of tumor cells and preventing the continued proliferation have been the primary means of clinical treatment of malignant tumors. The biggest problem faced by traditional treatment schemes, such as radiotherapy and chemotherapy, is that the sensitivity of tumor cells to radiotherapy decreases, and they are resistant to chemotherapy drugs. Therefore, some effective measures are urgently needed to reverse the insensitivity of tumor cells. Ferroptosis can help solve these problems. In light of such traditional treatment ideas as inducing tumor cell death, the answer given by ferroptosis is to cause tumor cell death by various mature ferroptosis inducers to prevent the infinite proliferation of cells. The FerrDb database shows 54 small molecular compounds that induce ferroptosis. For the problem of insensitivity to radiotherapy and chemotherapy, sensitivity can be enhanced by regulating the targets in the ferroptosis pathway. Therefore, the ferroptosis pathway is a valuable therapeutic target that deserves further investigation.

Esophageal cancer is one of the most common malignant tumors of the digestive tract in China. Due to its hidden incidence, most patients are already in the middle and late stages when they come to the hospital for treatment. Since the prognosis and survival rate of patients with early esophageal cancer is reasonable, improving the survival rate and quality of life of patients with mid and late-stage esophageal cancer has always been a complex problem that needs to be solved timely. Surgery is only limited to patients in the non-advanced stage and is less effective for patients in the advanced stage (Nakajima et al., 2016). Treating patients with advanced esophageal squamous cell carcinoma aims to alleviate symptoms and prolong survival. For a long time, cytotoxic drug therapy has been the primary means of chemotherapy, molecular targeted therapy, immunotherapy, and so on. However, the antitumor activity of traditional chemotherapy drugs such as cisplatin and 5-fluorouracil is still insufficient to meet the clinical needs. Despite the rapid development of molecular targeted therapy and immunotherapy, the individual differences of tumor suppressor patients and other factors need to be further studied (Hirano and Kato, 2019). Therefore, finding reliable and effective gene targets will be the future research direction. The metabolic pathway involved in ferroptosis contains many gene regulatory points, which indicates that it can provide us with abundant targets to design good experiments and provide practical new ideas for clinical treatment.

## Main regulatory mechanism of ferroptosis

### Production of lipid peroxide

The most typical morphological changes of ferroptosis are membrane rupture, Outer mitochondrial membrane rupture, reduction or deletion of mitochondrial cristae, *etc.* These membrane damage induced by ferroptosis is mainly due to the production of lipid peroxides. Therefore, the production of lipid peroxides can be broadly divided into iron ion absorption and Lipid peroxidation (Xie et al., 2016).

#### Iron ions

**FIGURE 2:**
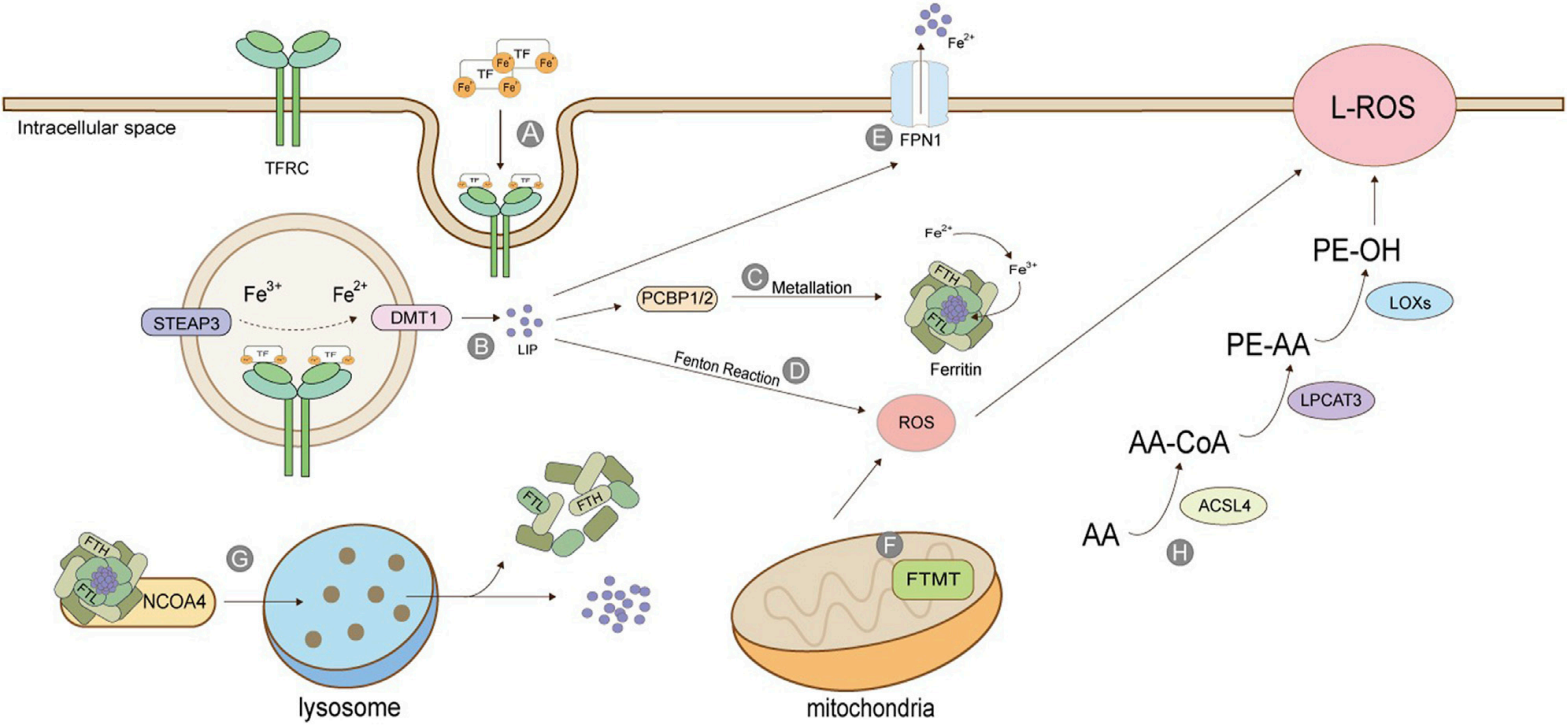
Production of Lipid Peroxide. **(A)** TF binds to TFRC and enters cells in the form of endosomes; **(B)** STEAP3 reduces Fe3+ to Fe2+, which is released into the cytoplasm by DMT1 to form labile iron pool; **(C)** Under the action of PCBP, the free iron is transferred to ferritin, which is composed of heavy chain FTH and light chain FTL, and has the activity of iron oxidase, oxidizing Fe2+ into Fe3+ and stores it; **(D)** Iron ion-mediated Fenton reaction; **(E)** Excessive iron ions are transported extracellularly by FPN1; **(F)** Mitochondrial ferritin; **(G)** Iron autophagy process; **(H)** Lipid peroxidation. The figure modified from (Dev and Babitt, 2017).

Iron ions in the human body can be divided into exogenous iron and endogenous iron. Exogenous iron comes from food and diet; generally, Fe3+, which is transported to the cell membrane by transferrin (TF) in serum, combines with transferrin receptor TFRC and enters the cell in the form of endocytosis (Kwon et al., 2015). The metal reductase STEAP3 contained in the endosome is responsible for reducing Fe3+ to Fe2+, which is then released into the cytoplasm under the action of divalent metal transporter (DMT1) to form labile iron pool (Figure 2). LIP has three destinations: most intracellular free irons transfer iron ions to ferritin by protein-protein interaction under the action of iron molecular chaperone PCBP1/2, also called metallization of ferritin. Ferrin is composed of heavy chain FTH and light chain FTL and has the activity of iron oxidase, which oxidizes Fe2+ to Fe3+ and stores it. The rest of cytoplasmic free iron undergoes a Fenton reaction with the cytosolic peroxide to mediate the production of ROS. Excess-free iron, in addition to these two uses, is excluded from the cell *via* the iron transporter 1 (FPN1) on the cell membrane (Anderson and Frazer, 2017; Dev and Babitt, 2017; Aversa et al., 2018; Di Sanzo et al., 2018) (Figure 2).

The release of stored iron, mainly the iron release process of ferritin *in vivo*, is an essential source of endogenous iron. Ferritin in mitochondria is FTMT, which is not ubiquitous and especially maintains the iron ion homeostasis in mitochondria. As iron ion is necessary for mitochondria to synthesize heme and iron-sulfur clusters, controlling iron ion content by FTMT is also critical (Mancias et al., 2014; Mancias et al., 2015). Ferritin is directed to lysosomes by a nuclear receptor coactivator (NCOA4). It degrades the protein part under the action of lysosomal enzymes, releasing iron ions and stabilizing iron content in cells. This process is also called iron autophagy. When iron autophagy is excessive, the concentration of iron ions in the body rises, inducing ferroptosis (Fuhrmann et al., 2020). Iron ion absorption is a prerequisite for ferroptosis, and many genes will also participate in the regulation of this process. For example, in lung cancer cells, the inhibition of cysteine desulphurase (NFS1) will up-regulate the expression of transferrin receptor TFRC, thus accelerating iron ion absorption and precipitating ferroptosis (Alvarez et al., 2017). Fanconi anemia complementary group D2 (FANCD2) is a nuclear protein involved in repairing DNA damage, which can regulate many genes of iron metabolism, such as FTH1, TF, TFRC, FPN1, and STEAP3 (Song et al., 2016). Serine/threonine kinase ATM indirectly regulates the expression of ferritin and iron transporter 1(FPN1) by holding the nuclear shift of metal-regulated transcription factor 1 (MTF1), thus controlling the process of ferroptosis (Chen et al., 2020).

#### Lipid peroxidation

The activation of polyunsaturated fatty acids (PUFA) is the first step and the critical regulatory point of ferroptosis, which needs the catalysis of acyl-coenzyme A long synthetase chain family member 4 (ACSL4) and lysophosphatidylcholine acyltransferase 3 (LPCAT3) (Mou et al., 2019; Cheng et al., 2020). ACSL4 catalysis is specific to fatty acyl (Kagan et al., 2017). Arachidonic acid (AA) and epinephrine (AdA) can produce arachidonic acid coenzyme A (AA-CoA) and epinephrine coenzyme A (AdA-CoA) under the action of ACSL4. The latter two can be inserted into membrane phospholipids under the act of LPCAT3 to be converted into PE -AA and PE-AdA, and finally oxidized to form lipid peroxides under the catalysis of lipoxygenase (LOXs) (Dixon et al., 2015; Sha et al., 2021) (Figure 2).

### Antioxidant system

The occurrence of ferroptosis originates from the imbalance of oxidation and antioxidant regulation. The oxidation reaction takes place in cells, and there are many sources of oxygen free radicals. As the powerful antioxidant system constantly counteracts these oxygen free radicals concurrently, generally, cells will not die because of excessive oxidative damage. To a large extent, the occurrence of ferroptosis can be attributed to the reduction of antioxidant capacity. When the antioxidant capacity of cells is weakened, even if the oxidative system of cells is not hyperactive, ROS produced by the general life activities of cells will cause the cells to die due to oxidative damage. Therefore, reducing antioxidant capacity is one of the critical mechanisms in ferroptosis. Glutathione (GSH) is one of the most abundant and powerful antioxidant molecules in cells and the leading force in the antioxidant system, whose metabolic disorder means the occurrence and development of ferroptosis. The systems XC - involved in GSH synthesis, GPx4 with GSH as an activating substrate, FSP1/CoQ10, DHODH, and others are presented below.

#### Cystine/glutamate antitransporter (System Xc-)

**FIGURE 3:**
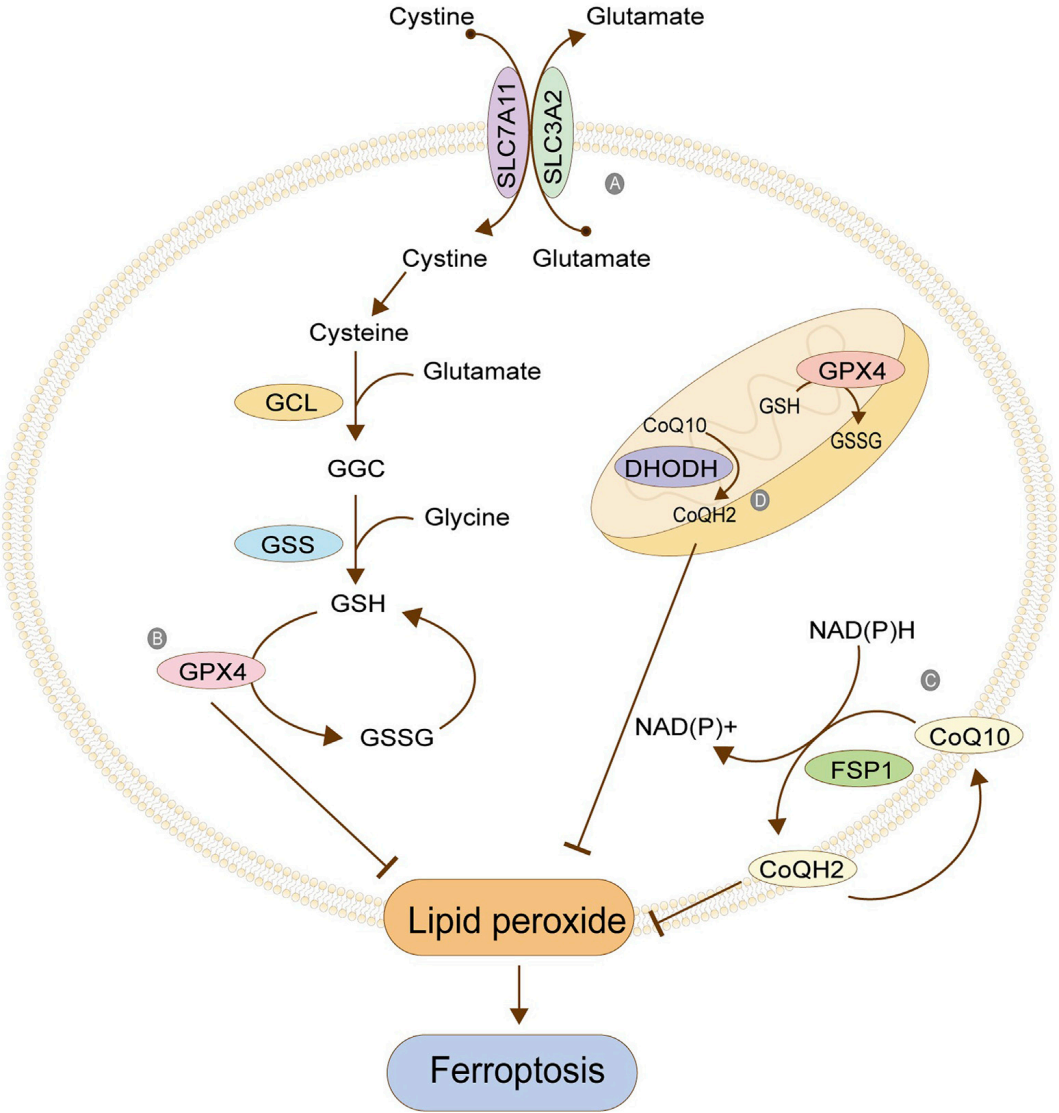
Antioxidant system. **(A)** Cystine/glutamate antitransporter; **(B)** Glutathione peroxidase 4; **(C)** FSP1/CoQ10; **(D)** Antioxidant system in mitochondria. The figure modified from (Anandhan et al., 2020).

System Xc- is a reverse transporter protein existing in the cell membrane, which transports glutamic acid out of the cell and cystine into the cell at the ratio of 1: 1 (Figure 3). This channel stops transporting when intracellular homocysteine and extracellular hyperglycemic acid are reached. This transporter is composed of light chain SLC7A11 (also called xCT) and heavy chain SLC3A2 (also called CD98), among which SLC7A11 plays a significant role in transport and accordingly determines the specificity of this system, and SLC3A2 is to stabilize SLC7A11. Cystine transported into cells is reduced to cysteine, one of the critical raw materials for synthesizing glutathione (GSH) and a rate-limiting precursor, which also acts as an antioxidant independently of GSH. GSH is synthesized from cysteine, glutamic acid, and glycine under the catalysis of γ -glutamylcysteine ligase (GCL) and glutathione synthetase (GSS), which is one of the most abundant antioxidant molecules in cells, also the number one molecule to resist ferroptosis. Its functions include maintaining intracellular redox balance, reducing oxygen free radicals, and maintaining the thiol state of the protein (Bassi et al., 2001; Dong et al., 2020; Lin et al., 2020; Koppula et al., 2021). Tables 1, 2 summarize the regulatory factors that regulate the two subunits in System Xc- respectively.

**TABLE 1:** Regulatory factors of SLC7A11.

Regulatory factor	The regulation of SLC7A11	Cancer
Metformin	downregulate	Breast cancer (Yang et al., 2021)
SOX 2	upregulate	Lung Cancer (Wang X. et al., 2021)
Lidocaine	downregulate	Ovarian and Breast Cancer (Sun D. et al., 2021)
P53	downregulate	Multiple cancer cell lines (Jiang et al., 2015)
Triptolide	downregulate	Head and neck cancer (Cai et al., 2021)
BAP1	downregulate	Kidney cancer (Zhang et al., 2019)
miR-27a-3p	downregulate	Non-small cell lung cancer (Lu X. et al., 2021)
Tanshinone IIA	downregulate	Gastric cancer (Guan et al., 2020)
ATF3	downregulate	Multiple cancer cell lines (Wang et al., 2020)
IMCA	downregulate	Colorectal Cancer (Zhang et al., 2020)
YAP/TAZ	upregulate	hepatocellular carcinoma (Gao et al., 2021)
TalaA	downregulate	colorectal cancer (Xia et al., 2020)
BECN1	downregulate	Multiple cancer cell lines (Song et al., 2018)
YTHDC2	downregulate	lung adenocarcinoma (Ma et al., 2021)
METTL3	upregulate	lung adenocarcinoma (Xu et al., 2022)
AKR1B1	upregulate	lung cancer (Zhang K. R. et al., 2021)
Levobupivacaine	downregulate	Gastric Cancer (Zhang K. R. et al., 2021)
Bavachin	downregulate	Osteosarcoma (Luo et al., 2021)
SFN	downregulate	Small-cell lung cancer (Iida et al., 2021)

SOX2, Stem Cell Factor 2; BAP1, H2A deubiquitinase; ATF3, activating transcription factor 3; IMCA, benzopyran derivative 2-imino-6-methoxy-2H-chromene-3-Carbothioamide; YAP/TAZ, transcription factor; TalaA, Natural compound, ferroptosis inducer; BECN1, beclin 1; YTHDC2, M6A reader; METTL3, methyltransferase-like 3; AKR1B1, aldo-keto reductase family 1 member B1; SFN, Sulforaphane.

**TABLE 2:** Regulatory factors of SLC3A2.

Regulatory factor	The regulation of SLC3A2	Cancer
ZEB1	downregulate	Ovarian Cancer (Cui et al., 2018)
IMiDs	downregulate	multiple myeloma (Heider et al., 2021)
miR-21	upregulate	hepatocellular carcinoma (Li et al., 2019)
AR-v7	upregulate	castration resistant prostate cancer (Sugiura et al., 2021)
DIO1	upregulate	renal cancer (Popławski et al., 2017)
PTPRJ	downregulate	lung cancer (D'Agostino et al., 2018)
IFNγ	downregulate	hepatocellular carcinoma (Kong et al., 2021)
MYCN	upregulate	neuroblastoma in mice (Gamble et al., 2019)
ADC	downregulate	triple negative breast cancer (Montero et al., 2022)
FZKA	downregulate	Non-Small Cell Lung Cancer (Zhao Y. Y. et al., 2022)

ZEB1, Zinc finger E-box-binding homeobox 1; IMiDs, immunomodulatory drugs; AR-v7, androgen receptor splice variant-7; DIO1, Type 1 iodothyronine deiodinase; PTPRJ, receptor protein tyrosine phosphatase; IFNγ, γInterferon-γ; MYCN, oncogene; ADC, antibody-drug conjugates; FZKA, Fuzheng Kang’ai decoction.

#### Glutathione peroxidase 4 (GPX4)

GPX4 is the core protein that regulates ferroptosis in cells, and it depends on GSH to achieve a substantial antioxidant effect. GSH is the activated substrate of GPX4, which transfers its sulfhydryl group to GPX4, allowing GPX4 to acquire antioxidant function and become oxidized glutathione (GSSG). The activated GPX4 reduces phospholipid peroxide PLOOH to the corresponding phospholipid alcohol PLOH, reduces phospholipid peroxide in the membrane, and removes oxygen free radicals, thus resisting the ferroptosis of cells (Figure 3). When GSH is depleted, GPX4 is inactivated, and oxidative damage occurs, leading to excessive lipid peroxidation and ferroptosis. GPX4, as an essential regulatory protein, is an important target of many ferroptosis inducers, the most typical of which is RSL3 (Maiorino et al., 2018; Bersuker et al., 2019; Xu et al., 2019; Zou et al., 2019). Table 3 summarizes other regulatory factors of GPX4.

**TABLE 3:** Regulatory factors of GPX4.

Regulatory factor	The regulation of GPX4	Cancer
RSL3	GPX4 Inactivation	Colorectal Cancer (Sui et al., 2018)
DMOCPTL	induced GPX4 ubiquitination	triple negative breast cancer (Ding et al., 2021)
FZD7	Indirectly upregulate GPX4	Ovarian Cancer (Wang R. et al., 2021)
Fin56	promote GPX4 protein degradation	bladder cancer (Sun Y. et al., 2021)
RB	GPX4 inactivation	colorectal cancer (Shen et al., 2021)
KLF2	transcriptional repression of GPX4	clear cell renal cell carcinoma (Lu Y. et al., 2021)
SFRS9	upregulate	Colorectal Cancer (Wang Y. et al., 2021)
circKIF4A	Indirectly upregulate GPX4	papillary thyroid cancer (Chen W. et al., 2021)
EBV	Indirectly upregulate GPX4	nasopharyngeal carcinoma (Yuan et al., 2022)
CREB	upregulate	lung adenocarcinoma (Wang Z. et al., 2021)
DHA	GPX4 Inactivation	glioblastoma (Yi et al., 2020)
GSTZ1	Indirectly downregulate GPX4	hepatocellular carcinoma (Wang Z. et al., 2021)
Metformin	Indirectly downregulate GPX4	breast cancer (Hou et al., 2021)
Apatinib	Indirectly downregulate GPX4	gastric cancer (Zhao et al., 2021)
Ketamine	Indirectly downregulate GPX4	Liver Cancer (He et al., 2021)

RSL3, ferroptosis inducer; DMOCPTL, a derivative of natural product parthenolide; FZD7, the Wnt receptor Frizzled-7; Fin56, ferroptosis inducer; RB, Resibufogenin; KLF2, Kruppel like factor 2; SFRS9, Serine and arginine rich splicing factor 9; circKIF4A, Circular RNA; EBV, Epstein-Barr virus; CREB, cAMP, response element-binding protein; DHA, Dihydroartemisinin; GSTZ1, Glutathione S-transferase zeta 1.

#### Ferroptosis-suppressor-protein 1 (FSP1)/coenzyme Q10 (CoQ10)

FSP1 is a GSH-independent ferroptosis regulator. When GSH antioxidant system is normal, the absence of FSP1 will still lead to lipid peroxidation. Therefore, it can be concluded that inhibiting lipid peroxidation mediated by FSP1 is parallel to the GSH system (Bersuker et al., 2019). FSP1 can be accurately localized on the cell membrane by its myoacylation structure. Its oxidoreductase domain is a significant part of ferroptosis, which is responsible for reducing the CoQ10 to reduced coenzyme Q (CoQH2) in the cell membrane. CoQ10 is a mobile lipophilic electron carrier that exists in all biofilms. CoQ10 can be recycled in the cell membrane, thus playing a more lasting role. Its reduction is completed by FSP1, and the required hydrogen is provided by NAD (P) H. The CoQ10 can be used as a free radical collecting antioxidant (RTA) to capture the oxygen free radicals in the cell membrane and further prevent the peroxidation of phospholipids in the cell membrane (Figure 3). The FPS1/CoQ10 system is parallel to GPX4 and System Xc, maintaining cell redox balance (Takahashi et al., 1993; Bentinger et al., 2007)

#### Antioxidant systems in mitochondria

GPX4 functions not only in the cytoplasm but also in the mitochondria. The antioxidant systems GPX4 and CoQ10/FSP1 in the cytoplasm are parallel. One exhibits down-regulation, and the other up-regulates its function, maintaining the overall antioxidant capacity of the cells. A similar phenomenon exists in mitochondria, where the GPX4 and the dihydroorotate dehydrogenase (DHODH) system maintain the balance (Figure 3). DHODH is a crucial enzyme involved in the *ab initio* synthesis of pyrimidine in prokaryotic and eukaryotic organisms, which is of great significance in cell chromosome replication. In addition, the enzyme is also related to ATP and ROS production. The function of DHODH is similar to that of the CoQ10/FSP1 system in cells in that it mainly helps the reduction of ubiquinone into panthenol in mitochondria to provide antioxidants to eliminate lipid peroxides in the mitochondrial membrane, thereby protecting the integrity of the mitochondrial membrane. The two mitochondrial systems, GPX4 and DHODH, are complementary. One system suffers from functional decline, and the other enhances its function, maintaining the antioxidant capacity (Mohamad Fairus et al., 2017; Mao et al., 2021).

#### Nuclear factor erythroid 2-related factor 2(NRF2)

**FIGURE 4:**
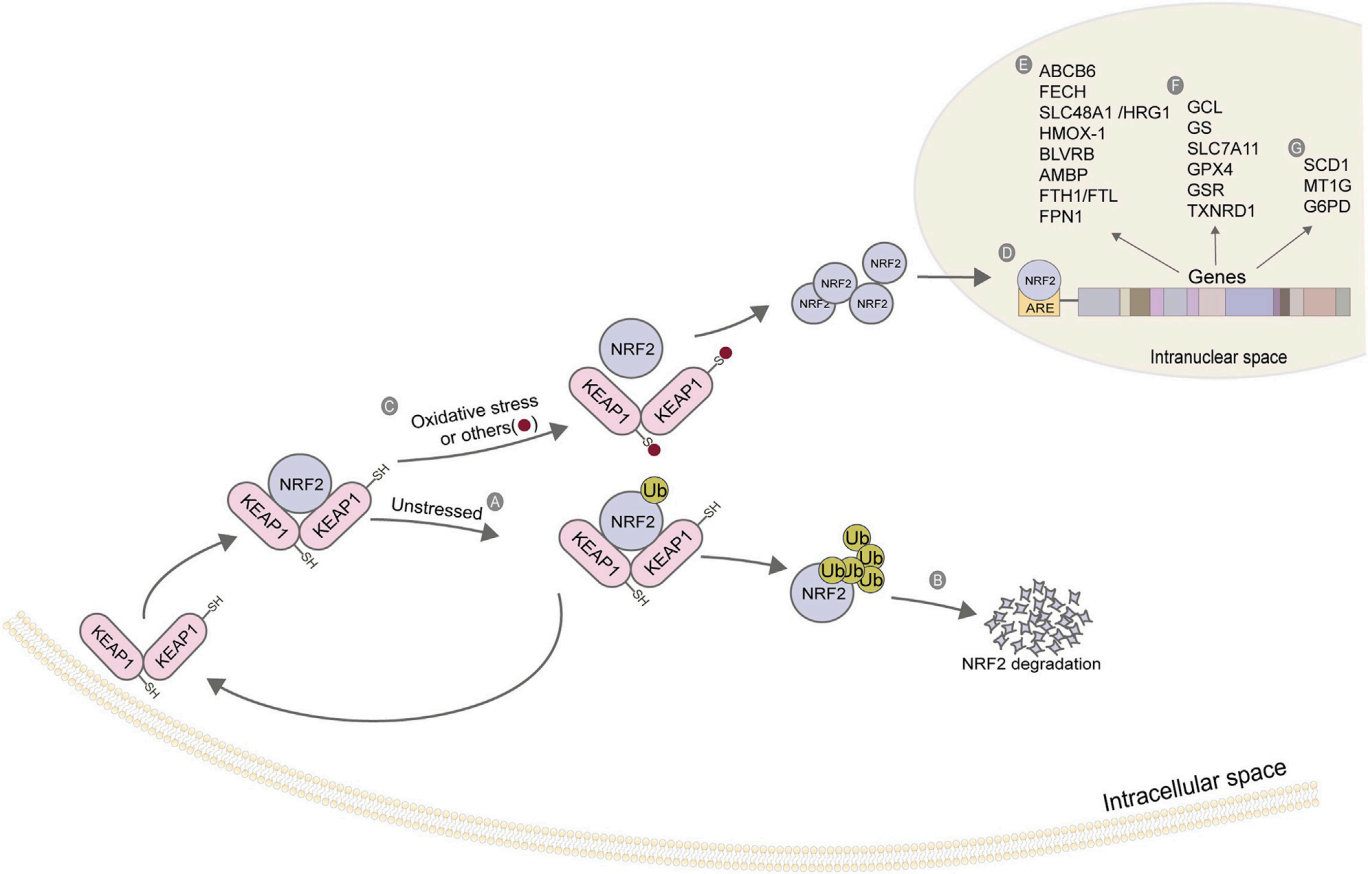
Action mechanism and regulatory genes of NRF2. **(A)** KEAP1 binds to NRF2, and NRF2 initiates ubiquitination under a non-stress state; **(B)** The ubiquitination of NRF2 strengthens its degradation; **(C)** Depolymerization of KEAP1-NRF2 complex under oxidative stress; **(D)** NRF2 enters the nucleus and binds to ARE; **(E)** Iron ion metabolism-related gene; **(F)** Glutathione metabolism-related genes; **(G)** Other related genes. The figure modified from (Kerins and Ooi, 2018).

As a stress-induced transcription factor, NRF2 plays a crucial role in maintaining the redox homeostasis of cells. Generally, NRF2 is at a low level in cells, which is maintained by Kelch-like ECH-related protein 1 (KEAP1). Under non-oxidative stress, KEAP1 binds to NRF2 to form a complex, which promotes the degradation of NRF2 by ubiquitination, thereby reducing the content of intracellular free NRF2. When cells are damaged by oxidation and other stress states, the KEAP1/NRF2 complex depolymerizes, releasing NRF2 (Kensler et al., 2007). A large amount of free intracellular NRF2 enters the nucleus. It binds with antioxidant response elements (ARE) located in the regulatory region to enhance the transcription of target genes (Figure 4), regulating their expression to rescue cells under stress (Ma et al., 2019). Most of the target genes regulated by NRF2 are involved in intracellular redox reactions, among which NRF2 regulates almost all the genes related to iron metabolism and glutathione metabolism. Table 4 summarizes the genes related to iron metabolism, glutathione metabolism, and other metabolism regulated by NRF2.

**TABLE 4:** Nrf2-regulated iron and glutathione metabolism related genes.

Gene abbreviation	Gene name	Involved metabolism
ABCB6	ATP binding cassette subfamily B member 6	Heme anabolism (Kerins and Ooi, 2018)
FECH	ferrochelatase	Heme anabolism (Kerins and Ooi, 2018)
SLC48A1/HRG1	Heme sensitive gene1	Heme catabolism (Kerins and Ooi, 2018)
HMOX-1	heme oxygenase	Heme catabolism (Kerins and Ooi, 2018)
BLVRB	biliverdin reductase B	Heme catabolism (Kerins and Ooi, 2018)
AMBP	alpha-1-microglobulin/bikunin precursor	Heme catabolism (Kerins and Ooi, 2018)
FTH1/FTL	ferritin heavy chain/light chain	iron storage (Kerins and Ooi, 2018)
FPN1	ferroportin1	iron mobilization (Kerins and Ooi, 2018)
GCL	glutamate–cysteine ligase	GSH anabolism (Lu, 2013)
GS	glutaminesynthetase	GSH anabolism (Lu, 2013)
SLC7A11	Solute Carrier Family 7	GSH anabolism (Dong et al., 2020)
GPX4	glutathione peroxidase 4	GSH reduction (Wang Q. et al., 2021)
GSR	Glutathione Reductase	GSH reduction (Hoffmann et al., 2017)
TXNRD1	Thioredoxin Reductase 1	GSH anabolism (Gao et al., 2020)
SCD1	Stearyl coenzyme A dehydrogenase-1	fatty acid metabolism (Huang et al., 2010)
MT1G	metallothionein (MT)-1G	MT1G-Nrf2/MT1G-P53-P21pathway (Sun et al., 2016)
G6PD	glucose-6-phosphate dehydrogenase	Pentose phosphate pathway (Dodson et al., 2019)

### P53 and ferroptosis

P53 is a well-known cancer suppressor gene, which mainly achieves its arrest action during cancer development through cell cycle arrest, cell senescence, and apoptosis. Recently, more and more studies have shown that P53 has a close correlation with and a potent function in a novel death mod, ferroptosis, apart from its classical role mentioned above, which involves several vital molecules in the ferroptosis pathway, such as SLC7A11, alox12, GPx4 and so on.

These results suggest that P53 exerts a tumor suppressor effect by regulating the cell cycle and mediates ferroptosis by regulating ferroptosis-related molecules. SLC7A11 is one of the vital ferroptosis inhibiting molecules, which provides essential components for glutathione synthesis by transporting cystine, thus inhibiting the occurrence of ferroptosis. Studies have shown that P53 binds to the promoter region of SLC7A11 in many human cancer cell lines through experiment verification, thereby down-regulating its protein expression, reducing the inhibition of tumor cells on ferroptosis, and finally promoting ferroptosis. In addition, it was surprising to find in these studies that the mutant P53^3KR^ (an acetylation–defensive mutant) lost the function of cell cycle arrest but ultimately retains the process of down-regulating SLC7A11. This study provides us with a new powerful function of P53 in tumor inhibition (Jiang et al., 2015). ALOX12(Arachidonate-12-Lipoxygenase) is a member of the lipoxygenase family, and its role in ferroptosis is mainly to realize lipid peroxidation (shown in Figure 2), which is also the initial step of ferroptosis. Studies have shown that ALOX12 is an essential molecule in ferroptosis mediated by P53. It was also found that the knock-out of Alox12 fails to affect the protein expression levels of P53 and the downstream targeted genes of P53, but it can eliminate P53-mediatedferroptosis (Chu et al., 2019). The regulatory relationship between P53 and GPX4 was reported by (Chen et al., 2021a). In the vascular endothelium induced by oxygen and glucose depletion (OGD) combined with high glucose (HG), P53 was regulated by the LncRNA Meg3 and accordingly unregulated. Then the highly expressed P53 entered the nucleus and combined with the regulatory region of GPX4 to down-regulate the expression of GPX4, finally preventing the scavenging effect of GPX4 on lipid peroxide. This study revealed the mechanism of LncRNA Meg3 inducing ferroptosis in vascular endothelial cells through the P53-GPX4 axis.

In 2021, studies reported the related mechanism of calcium-independent phospholipase iPLA2β inhibiting ferroptosis. iPLA2β has the detoxification effect of lipid peroxides, and IPLA2β can inhibit ROS-induced p53-driven ferroptosis in tumor cells. And p53-driven ferroptosis is independent of GPX4. The team first demonstrated that p53-driven ferroptosis independent of GPX4 in response to ROS-induced ferroptosis and subsequently revealed that loss of iPLA2β expression significantly increased the sensitivity of cancer cells to p53-dependent ferroptosis in response to ROS-induced ferroptosis. Although both GPX4 and iPLA2β inhibit ferroptosis by lipid peroxide detoxification, this study reveals the differences in the working principles of IPLA2β and GPX4 (Chen D. et al., 2021). Based on the above results, we can understand the powerful function of P53 in regulating ferroptosis. Liu et al. divided the mechanism of P53 regulating ferroptosis into two categories: canonical (GPX4-dependent) and non-canonical (GPX4-independent) and gave a very comprehensive summary. P53 by cell metabolism, iron ion, lipid metabolism, glutathione, and the synthesis of ROS aspects of regulation and control ferroptosis, in most cases, P53 promotes ferroptosis. As a result, the authors have also proposed several schemes to target P53 to treat tumors and neurodegenerative diseases through the ferroptosis pathway (Liu and Gu, 2022). Both the traditional tumor suppressor mechanism of P53 (cell cycle arrest, *etc.*) and the non-traditional tumor suppressor mechanism (promoting ferroptosis, *etc.*) are sufficient to make P53 the focus of targeted drug development. In the future, our research should focus on developing new targeted drugs using p53-mediated ferroptosis and combing traditional cancer therapies with p53-mediated ferroptosis, to propose therapeutic strategies with better efficacy. More and more studies are devoted to discovering the unconventional tumor-inhibiting mechanism of P53. To our surprise, many pieces of evidence demonstrate that P53 can achieve its tumor-inhibiting effect through ferroptosis. Based on this, we can continue to improve the clinical research of P53-mediated ferroptosis in the treatment of human cancer and further explore other potential mechanisms of p53.

## Study on ferroptosis in esophageal cancer

Esophageal cancer is the sixth most common cause of cancer death in the world, which can be divided into esophageal squamous cell carcinoma (ESCC) and esophageal adenocarcinoma (EAC) from the pathological point of view. Nearly 90% of esophageal cancer patients in the world are ESCC patients. The most unfortunate for esophageal cancer is that it is already in the middle and late stages with a poor prognosis at the time of diagnosis. The 5-year survival rate is less than 20% in developed countries and less than 5% in developing countries. Thus, early screening, diagnosis, and treatment for ESCC patients are critical (Jemal et al., 2011; Arnold et al., 2015; Codipilly et al., 2018). Clinical efforts have been devoted to finding marker molecules representing the early stage of cancer, whose characteristic changes can become a reliable signal, thus playing a predictive role and providing robust evidence for early screening of esophageal cancer patients.

In essence, ferroptosis is an oxidative damage process induced by the imbalance of oxidation and antioxidant systems. In 2014, Sehitogullar et al. detected the serum of 33 patients with ESCC after primary cancer resection to catch the oxidative stress level of ESCC patients. The results showed that the activities of glutathione peroxidase (GPXs), glutathione reductase (GR), and superoxide dismutase (SOD) in the ESCC group were lower than those in the control group. At the same time, the level of malondialdehyde (MDA) was significantly higher than that in the control group. This data set suggested that ESCC patients have a high level of oxidative stress (Sehitogullari et al., 2014). In 2020, Xiong et al. mentioned in their research that the expression level of SLC7A11 in cystine/glutamic acid transporter was significantly increased in ESCC patients, and it was verified by data mining analysis that SLC7A11 was a differentially expressed gene, and the survival prognosis of its highly expressed patients was poor. In addition, they preliminarily speculated that SLC7A11 might participate in ferroptosis through P53 and ROS metabolic pathways through functional enrichment analysis (Xiong et al., 2020). In 2019, Wang et al. proposed that the mutation of isocitrate dehydrogenase 1(IDH1), a key enzyme in the tricarboxylic acid cycle, could enhance the sensitivity of ESCC KYSE170 cells to Erastin (ferroptosis inducer). Specifically, D-2- hydroxyglutaric acid, a metabolite produced by mutant IDH1 in tumor cells, reduces the expression of antioxidant GPX4 in tumor cells, thus enhancing the ferroptosis sensitivity induced by Erastin (Wang et al., 2019). Jiang et al. found that the expression level of DnaJ/Hsp40 homolog B subfamily 6 (DNAJB6) in ESCC tissue was lower than that in normal esophageal cancer tissues, and its expression level was negatively correlated with lymph node metastasis. The cancer inhibition effect of the DNJB6 was verified by *in vivo* and *in vitro* experiments. Through further mechanism research, they were surprised to find that the overexpression of DNAJB6 gene was accompanied by a significant decrease in GPX4 and p-AKT protein levels. At the same time, the increase in lipid peroxidation level and the characteristic changes of ferroptosis in mitochondria were detected. Accordingly, they proposed for the first time compelling evidence that overexpression of DNAJB6 increased ferroptosis in esophageal cancer cells by down-regulating GPX4 (Jiang et al., 2020).

NRF2 is a well-known transcription factor that regulates antioxidant system proteins. In ESCC, NRF2 still upregulates the expression of SLC7A11 as its role as a transcription factor. The upregulated SLC7A11 is beneficial to the synthesis of intracellular GSH, further enhancing the resistance to ferroptosis. The inhibition of the ferroptosis pathway instead promotes the radioresistance of ESCCs (Feng et al., 2021). The team of Chen et al. identified a new neuropeptide-related ferroptosis pathway and found that neuropeptide LGI1 receptor ADAM23 not only inhibited the proliferation and migration of ESCCs but also upregulated the ferroptosis pathway. The mechanism may be that overexpressed ADAM23 promotes ferroptosis by consuming GPX4, SLC3A2, and SLC7A11. The up-regulation of ADAM23 is complete by the upstream ARHGEF26-AS1 (LncRNA) regulating miR-372-3p, positively regulating ADAM23 (Chen et al., 2021b).

### Ferroptosis of esophageal cancer cells induced by anticancer therapy

Radiotherapy has always been one of the effective treatments for patients with esophageal cancer, whose principle is DNA double bond breakage and cell cycle stagnation, which is well known. Recently, Lei et al. discovered that after radiotherapy, ROS level and ACSL4 level in FOL-1 cells of esophageal adenocarcinoma were significantly up-regulated, both of which were prerequisites for ferroptosis. At the same time, FOL-1 cells exhibited an adaptive response to radiotherapy by up-regulating the levels of SLC7A11 and GPX4. That is, radiotherapy can up-regulate both the promotion and the inhibition pathways of ferroptosis in FOL-1 cells. On these grounds, they further used inhibitors of SLC7A11 and GPX4 simultaneously with radiotherapy. They found that the sensitivity of FOL-1 to radiotherapy was improved, thus preliminarily revealing the close relationship between the ferroptosis pathway and radiotherapy (Lei et al., 2020). Terpenoids have attracted the attention of researchers because of their excellent pharmacological effects, such as anticancer, anti-inflammation, anti-oxidation, and so on (Ku and Lin, 2013). And Oridonin (Ori) is one of them. Zhang et al. verified Ori’s anticancer activity to inhibit esophageal cancer cells’ growth in their experiments and proposed the relationship between the anticancer activity of Ori and ferroptosis. After treating of ESCC cell TE-1 by Ori, intracellular iron ions, malondialdehyde (MDA) levels, and ROS were significantly dose-dependent. In addition, metabonomics analysis showed that Ori significantly inhibited the γ-glutamyl cycle of TE-1 cells, decreasing intracellular glutathione synthesis and thus inducing the ferroptosis of cells (Zhang J. et al., 2021). The anticancer activity of isopropyl lactone in many cancers was confirmed, and its effect in esophageal cancer has been initially proposed in the study of Lu et al. They detected the impact of isopropyl lactone on inhibiting invasion and migration in ESCC Eca109 cells, which was mediated through such means as apoptosis and ferroptosis. After the isopropyl lactone treatment, the isopropyl lactone caused the apoptosis of cells by up-regulating the external apoptosis pathway of death receptor 5 (DR5) to activate cells and, simultaneously, caused ferroptosis by increasing intracellular ROS levels. To verify this, they treated Eca109 cells with ferroptosis inhibitor combined with isopropyl lactone and found that the cell death was saved and the cell viability was improved (Lu et al., 2018). Pentaaminolevulinic acid (5-ALA), a natural amino acid, is widely used in cancer treatment. Current research shows that 5-ALA induces ferroptosis through glutathione peroxidase 4 (GPX4) and HMOX1, and has an anti-tumor effect in ESCC (Shishido et al., 2021).

Preventing the continued proliferation of cancer cells and promoting the death of cancer cells is one of the primary purposes of anti-cancer treatment. As a cell death mode characterized by oxidative damage, ferroptosis has become an essential link in many cancer treatment drugs and means. In addition to their classical anti-tumor effects, the radiotherapy and classical anti-cancer drugs mentioned above have also been confirmed to promote ferroptosis of cancer cells by increasing ferroptosis-inducing factors such as intracellular iron ions and ROS, inhibiting GPX4 expression, reducing GSH content and the like, which is a beneficial and surprising discovery. According to these findings, the existing clinical anti-cancer treatments can be further enhanced, for example, chemoradiotherapy combined with ferroptosis inducer treatment which increases the mortality of cancer cells and also increases the sensitivity of cancer cells to chemoradiotherapy; the design of targeted drugs against ferroptosis inhibitory factors combined them with classical anticancer drug therapy to improve the ability of anti-cancer medications to promote ferroptosis of cancer cells.

### Prediction of the prognosis of esophageal cancer patients by ferroptosis-related genes (FRGs)

Lu et al. systematically identified FRGs TFRC, ATG5, ENPP2, SCP2, MAPK1, and PRKAA1 as valuable genes for predicting the prognosis of ESCC, among which SCP2, MAPK1, and PRKAA1 were identified as independent prognostic genes (Lu T. et al., 2021). Similarly, they identified ALOX12, ALOX12B, ANGPTL7, DRD4, MAPK9, SLC38A1, and ZNF419 as prognostic FRGs in patients with ESCC, among which the expression of ALOX12, ANGPTL7, DRD4 and MAPK9 in ESCC was significantly decreased. In contrast, the expression of SLC38A1 and ZNF419 was significantly increased, but the expression of ALOX12B was not significantly different. On this basis, the researcher selected SLC38A1 with noticeable expression difference to further explore its influence on the proliferation and migration of ESCC cells and finally concluded that SLC38A1 promoted the proliferation and migration of ESCC cells (Song et al., 2021); Ye et al. identified SRC, FADS2, GLUD1, POLG, ANO6, SLC2A6, PTGS2, ALOXE3, *etc.* (Ye et al., 2021); Zhao et al. identified six FRGs with prognostic value in 112 ESCC samples (Zhao M. et al., 2022). Among them, the expressions of PRNP, SLC3A2, SLC39A8, and SLC39A14 were negatively correlated with the prognosis of ESCC patients, while the expressions of ATP6V0A1 and LCN2 were the opposite. They also evaluated the infiltration of immune cells, and the results showed that the expressions of ATP6V0A1, SLC39A14, SLC39A8, and LCN2 were positively associated with the infiltration of B cells. In contrast, the expressions of SLC3A2 and PRNP were negatively correlated with the infiltration of B cells. The same study also reported a significant correlation between 74 FRGs (Zhao Y. Y. et al., 2022) and the risk score of esophageal cancer patients, among which 32 genes were positively correlated with the risk score, with the top five genes being HIC1, ACSL3, NNMT, ANO6 and CDO1, and 42 genes were negatively correlated with the risk score, with the top five genes being HAMP, ALOX12B, MAFG, HILPDA and DUOX1 (Shi et al., 2021).

Many ferroptosis-related genes mentioned above have different prognostic values for esophageal cancer patients, even some of which can become independent prognostic factors. The deep exploration and further improvement of these genes can provide early signals for the occurrence and development of esophageal cancer, thereby helping the clinic to take preventive and therapeutic measures earlier. In addition, these ferroptosis-related genes with prognostic values are significantly related to the tumor immune microenvironment. Further, combined with the correlation of immune check inhibition points, this significant correlation can be used clinically to design a more reliable immunotherapy protocol to tap the potential of prognostic ferroptosis-related genes in immunotherapy of esophageal cancer.

### Non-coding RNA regulating FRGs in esophageal cancer cells

CircPVT1 is a circular non-coding RNA and a crutial regulatory factor in the pathogenesis of ESCC. For example, it can enhance the malignant phenotype of ESCC by regulating the miR-4663/Pax and PPAR axis, including the proliferation and invasion of ESCC cells (Zhong et al., 2019). A recent study showed that circPVT1 was significantly upregulated in 5- fluorouracil-resistant ESCCs, and the knock-down of circPVT1 significantly reduced the expression levels of GPX4 and SLC7A11, which indicated that the overexpression of circPVT1 could upregulate the expression of GPX4 and SLC7A11, thus enhancing the ferroptosis resistance and chemotherapy drug resistance of cancer cells. In addition, their results also showed that CircPVT1 regulated the chemosensitivity of ESCCs through miR-30a-5p/FZD3 axis (Yao et al., 2021). Methylene selenite (MSA) is a selenium substituted compound which realizes the nuclear transfer of NRF2 by down-regulating the expression of Keap1 in ESCCs. The accumulation of NRF2 in the nucleus will strengthen its binding with antioxidant response elements (ARE), thus upregulating the expression of antioxidant-related genes. Further studies showed that miR-200a mainly mediated MSA’s down-regulation of Keap1 expression. MSA firstly upregulates the expression of miR-200a, while Keap1 is the direct downstream target of miR-200a. Highly expressed miR-200a inhibits the expression of Keap1, resulting in increased nuclear metastasis of NRF2 and further enhancing the antioxidant activity of cells (Liu et al., 2015). Keap1 is also a direct target of miR-432-3p, which directly binds to the regulatory region of Keap1 and then down-regulates its expression, thereby positively regulating NRF2. Besides, miR-432-3p is over-expressed in primary ESCC, which is closely related to the decreased sensitivity of chemotherapeutic drugs such as cisplatin (CDDP) (Akdemir et al., 2017). Noncoding RNAs directly targeting NRF2 include miR-507 (Yamamoto et al., 2014) and MicroRNA-153-3p (Zuo et al., 2020). miR-507 inhibits the expression of NRF2 through direct targeting, thus inhibiting the carcinogenesis pathway mediated by NRF2. The author found that miR-507 was low in ESCC, thus losing its inhibitory effect on NRF2. To further determine the target genes regulated by NRF2, the expression of eight NRF2 transcription target genes which were obviously inhibited in ESCCs transfected with miR-507 was detected, which further determined the target genes regulated by NRF2, including SLC7A11; MiR-153-3p can inhibit the proliferation of food cells and enhance cisplatin resistance by down-regulating the expression of NRF2 in Eca-109 cells.

Non-coding RNA, as an important regulatory molecule in gene expression, regulates disease occurrence, development, and outcome. With the molecular mechanism of the ferroptosis pathway becoming more apparent, more and more related genes have been confirmed. The discovery and research of the upstream regulation mechanism of these genes have become a hot spot, among which non-coding RNA is widely studied. The non-coding RNA listed above affects the proliferation, invasion, and metastasis of esophageal cancer cells by regulating the ferroptosis pathway during the occurrence and development of esophageal cancer. On these grounds, corresponding detection means can be developed to detect the non-coding RAN with biomarker value in esophageal cancer patients at different stages, which can be applied to clinical diagnosis and staging. With its role in regulating the ferroptosis pathway, small molecular compounds that promote or prevent the combination of non-coding RNA and target genes can be developed to intervene in the ferroptosis process in esophageal cancer cells.

## Summary and prospect

As the prognosis of early esophageal cancer patients is good, the early diagnosis of esophageal cancer is vital. For mid and late-stage patients, relying on surgery alone is infeasible, and the best treatment method is to combine chemoradiotherapy; however, radiotherapy insensitivity and chemotherapy drug resistance are often one of the main reasons that affect the treatment effect. Presently, the mechanism of ferroptosis in esophageal cancer is extensively studied, including such issues as some ferroptosis inducers combined with chemotherapy drugs have more obvious efficacy than that of the two drugs alone but also improve the sensitivity of chemotherapy drugs. Many ferroptosis-related genes have been verified as prognostic genes of esophageal cancer. The changes in their expression levels in different stages of esophageal cancer patients can provide beneficial information for clinics and robust evidence for clinicians to accurately diagnose and stage. In addition, by up-regulating or down-regulating the essential regulatory point genes in the ferroptosis pathway, the ferroptosis of esophageal cancer cells can be promoted utilizing elevated levels of intracellular iron ions, ROS, and lipid peroxidation, thereby achieving tumor inhibition. Secondly, combined with the current hot immune checkpoint treatment, the correlation between the differentially expressed genes related to ferroptosis and each immune checkpoint in esophageal cancer was analyzed to find out the genes with significant correlation for further design of immune checkpoint related immunotherapy.

The research on the mechanism of ferroptosis is deepening, but there are still more elaborate and complex networks of signal pathways waiting for us to explore. In future research, we need to reveal and analyze the molecular mechanism of ferroptosis in cells more comprehensively from metabolism, immunity, and genetics perspectives. There is relatively little research on the mechanism of ferroptosis in esophageal cancer. Its research mainly focuses on predicting the prognosis of esophageal cancer patients, providing us with many potentially valuable genes. Next, the focus of our study should be to find out the most valuable FRGs from the data supplied by bioinformatics for in-depth mechanism research and dig out more mechanisms for regulating ferroptosis. The research results will provide clinical treatment strategies and powerful evidence in pharmacology to develop corresponding ferroptosis inhibitors or inducers. For cancer patients who have developed chemotherapy drug resistance, applying the ferroptosis mechanism in treatment will bring them new hope.
